# A Large Left Ventricle Myxoma: Presenting with Epigastric Pain and Weight Loss

**DOI:** 10.1155/2016/9018249

**Published:** 2016-12-20

**Authors:** Solmaz Fakhari, Eissa Bilehjani

**Affiliations:** ^1^Department of Anesthesiology, Tabriz University of Medical Sciences, Madani Heart Hospital, Tabriz, Iran; ^2^Department of Cardiovascular Anesthesia, Tabriz University of Medical Sciences, Madani Heart Hospital, Tabriz, Iran

## Abstract

Cardiac myxomas are the most common benign tumors found in the heart. They usually appear in the left atrium. Those originating from the left ventricle (LV) are rare. Although clinical presentation may vary, dyspnea and embolism are the most commonly reported symptoms. In the present case study, a 27-year-old woman with a large myxoma originating from the left ventricular free wall is studied. She had atypical complaints, mainly epigastric discomfort, nausea, vomiting, and anorexia. She was hospitalized for acute abdomen, but subsequent investigations revealed a large myxoma that fully filled the LV and severely compromised the flow of the aortic and mitral valves. After successful emergency tumor resection, all symptoms disappeared. The uncommon presentation caused by these tumors is discussed in this study.

## 1. Introduction

Cardiac myxomas are the most common tumors found in the heart [1, 2]. Although myxomas are benign tumors, they can cause significant morbidity and mortality [1, 3]. Tumors are found mainly in the left atrium but can grow in any chamber [2]. Generally, left ventricular (LV) myxomas are extremely rare. Myxomas usually originate from the septum and appear as systemic embolization, episodes of syncope as the consequence of arrhythmia or LV outflow tract obstruction [4–10], and systemic symptoms resulting from interleukin-6 production by tumor cells. In rare case reports, the tumor originates from the posterior [7] or lateral wall of the LV [4, 10]. Although echocardiography has improved tumor detection, diagnosed left ventricular myxomas are still rare. Among various clinical presentations, syncope and dyspnea are the most common reported symptoms [7]. Cardiac myxomas may cause emboli to the central nervous system and elsewhere in the vascular tree [10, 11]. When patients present with nonspecific symptoms, the tumor may be misdiagnosed for a long time until the onset of more significant embolic complications, such as a stroke with functional impairment [6]. The current inquiry presents and discusses a patient with a large left ventricular myxoma whose main complaints were epigastric discomfort, nausea, vomiting, and weight loss.

## 2. Case Presentation

**Figure 1:**
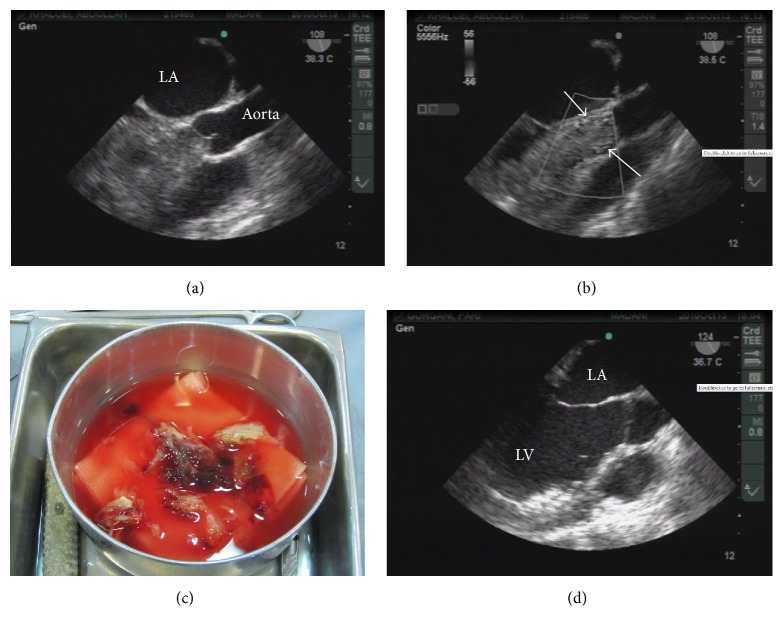
The large left ventricle (LV) mass on intraoperative transesophageal echocardiography (mid esophageal aortic valve, long axis view). Please note its position during diastole (a), systole (b), left ventricular chamber after its removal (d), and limited blood flow around it in color Doppler imaging (b). The mass was classified as a myxoma in histopathologic examination (c).

The patient was a 27-year-old woman (weight = 48 kg, height = 171 cm) admitted to a general hospital with primary diagnosis of acute abdomen because of acute epigastric pain, nausea, and vomiting. She had a history of activity-induced dyspnea and chest discomfort for 18 months. Anorexia, weight loss (approximately 10 kg), and episodes of flushing and syncope while standing were added to her symptoms at the past 3 months. In spite of a few medical visits at the past year, there was not any definitive diagnosis. Episodic epigastric pain referred to the right shoulder lasted for 15 min; nausea and vomiting were reported as the main symptoms in the last two weeks. In the physical examination, she was cachectic, acyanotic, ill, and tachypneic with respiratory distress. Her vital signs were as follows: a regular heart rate 100 beat/min, blood pressure 80/45 mmHg, axillary temperature 36.7°C, and respiratory rate 24 per minute. Respiratory sounds decreased in the lower half of the lungs. There was a muffled first heart sound with a moderate holosystolic murmur in the apex. There was not either guarding or tenderness on abdomen or abdominal organomegaly. Laboratory data were normal except for a mild increase in ESR. The chest X-ray revealed increased chest/thoracic ratio and bilateral plural effusion. A thoracic and abdominal CT scan revealed cardiomegaly with a large mass (45*∗*32 mm) in LV and bilateral pleural effusion without any pathologic findings in the abdomen. Transthoracic echocardiography (TTE) was done as a final diagnostic modality and reported a completely occupied LV with a large nonhomogenous mass protruding into aortic valve and severely compromised ejection fraction (0.15), limited valve opening, and flow through mitral and aortic valves. Preoperative lab tests were showing the normal values for blood, renal, and hepatic function tests. She was transferred to our heart hospital for an emergency surgery. With invasive arterial blood pressure monitoring, anesthesia was induced using midazolam 10 mg, ethomidate 10 mg, fentanyl 100 *μ*g, and cisatracurium 10 mg. Intraoperative transesophageal echocardiography (TEE) revealed severely decreased LV function; LV chamber was totally filled with a large mass (76*∗*44 mm), compromised mitral valve opening (moderate regurgitation), and a systolic protrusion of the mass to the aorta (approximately 1.5–2 cm). On the TEE, it seemed that the mass was pedunculated, originating from the LV lateral wall (Figure 1). Using cardiopulmonary bypass (CPB) and a transmitral and aortic root approach, the huge multilobulated jelly-like mass was resected piece-by-piece. It had been attached to the apical-lateral wall. After deairing of the heart, CPB discontinued using dobutamine infusion 10 *μ*g/kg/min with a sinus rhythm. Postbypass TEE revealed a moderate MR with severe LV systolic dysfunction (ejection fraction 0.10–0.15). Postoperative period was without any complication, and almost all the complaints were resolved rapidly. Renal, hepatic, and routine blood tests were within normal limits postoperatively. A blood sample was prepared and sent to a referral regional lab in order to measure the serum IL-6 concentration, in the second postoperative day. The patient left the ICU and hospital on the third and seventh postoperative days, respectively, in a good clinical condition. TTE on the third postoperative day showed a moderate eccentric mitral regurgitation (MR) with LVEF of 0.30. Pathologic examination confirmed myxoma diagnosis. Three months later, TTE showed a normal heart (LVEF 0.45) with only mild MR. Lab report presented the postoperative serum IL-6 concentration (assayed by Bender MedSystem, Austria) as a normal value (2.98 pg/mL).

## 3. Discussion

Primary cardiac tumors are extremely rare with a prevalence of 0.001% to 0.3% upon autopsy [1, 2, 12]. In all age groups, benign primary cardiac tumors are more common compared with malignant tumors (75%), and the majority of benign tumors are myxomas [1, 2]. Myxomas originate from the endocardium, usually the interatrial septum [2, 4]. According to previous studies, most myxomas grow in the left atrium (70–90%), and only a small percentage (15–20%) is found in the right atrium. Tumors may rarely originate from the right or left ventricles [13].

Clinical symptoms are usually nonspecific and may not lead to tumor diagnosis for a long period. Until 60 years ago, these tumors were considered a postmortem diagnostic event [12]. Along with improvements in diagnostic modalities, ante-mortem diagnosis and surgical resection of the tumors have become more feasible [12]. The most simple and effective tool for its diagnosis is echocardiography. This technique precisely locates the tumor and defines its extension [12, 14]. The clinical signs and symptoms of the tumor are atypical and vary greatly [14, 15]. They may be related to the following mechanisms:

- The tumor mass can interfere with valve function mechanically and obstruct intracardiac blood flow.
- Local invasion of the tumor can lead to arrhythmias or pericardial effusions with tamponade.
- Pieces of the tumor can cause systemic or pulmonary embolization on the left or right side of the heart, respectively.
- The tumor may produce and release a few substances into the circulatory system which may be responsible for inflammatory or autoimmune problems (as interleukin 6).

Some tumors (4%) produce no symptoms and are diagnosed only as an accidental finding [1].

The long list of reported symptoms and signs includes chest pain, dyspnea, orthopnea, fever, malaise and fatigue, weight loss, pancreatitis [9], cough, palpitation, cyanosis and clubbing, Raynaud's phenomenon, arthralgia, myalgia, muscle weakness, loss of hair, dizziness, fainting, aphasia, peripheral embolism [16], syncope, transient ischemic attack (TIA), cerebrovascular accident (CVA) [10–12, 17], sudden cardiac death [5, 12, 18], and heart failure [19]. These symptoms may accompany the change in body position. Although the cause of constipation is not well known, it may disappear rapidly after tumor resection [12]. Robert et al. reported a case of left ventricular myxoma presented with sudden transient visual loss, frequent palpitations, and dizziness episodes [10].

Recent studies suggest that myxomas produce and release interleukins into the blood circulatory system, which may be responsible for the wide spectrum of systemic inflammatory or autoimmune problems [9]. Symptoms such as constipation, nausea, vomiting, and anorexia may be related to the production of interleukin 6 (IL-6), a principal mediator of the acute phase protein response [9, 13]. The type and duration of clinical presentation of myxoma depends on tumor size, location, mobility, growth rate, and histopathologic features [4, 7]. The current study case encountered a cluster of symptoms including epigastric pain, anorexia, nausea, vomiting, and weight loss, which may easily mislead the general medical practitioners to come up with a misdiagnosis. If the long-term history of the patient had been considered, the certain markers of low cardiac output state (activity-induced fatigue, dyspnea and chest discomfort, episodes of flushing, and syncope upon standing) would have been diagnosed in the patient. Thus, the heart mass could be discovered easily by searching logical explanations for all of the signs. Mitral regurgitation can cause dyspnea [14], and LV inflow and outflow obstruction can cause activity induced fatigue, chest discomfort, anorexia, weight loss, syncope upon standing, nausea, and vomiting. Emboli to systemic circulation can mimic acute abdomen syndrome, nausea, and vomiting and ultimately production of interleukin 6 can cause flushing, elevated ESR, and fever. Accordingly, the study patient did not certainly have any unusual presentation. She had various symptoms for a long period, but because of the absence of a clinical suspicion, her problem remained undiagnosed for nearly 18 months. According to previous reports, LV myxomas usually grow from the septal endocardium [18], and there are only two case reports of growth from an LV free wall [10]. Thus, the study case is a new and rare case of myxoma grown from the LV apical-lateral wall.

## 4. Conclusion

Left ventricular myxoma may produce various systemic and nonspecific symptoms including anorexia, nausea/vomiting, weight loss, flushing, syncope, and abdominal discomfort. The basic action for its diagnosis is a good clinical suspicion and judgment, and then a conventional echocardiographic exam can usually confirm the diagnosis.
